# Extracellular vesicle-associated transcriptomic and proteomic biomarkers show in vitro potential for vandetanib treatment monitoring in anaplastic thyroid cancer

**DOI:** 10.1038/s41598-025-18319-w

**Published:** 2025-09-12

**Authors:** Christian Grätz, Prashant Changoer, Dapi Menglin Chiang, Johannes Kersting, Martin Jaeger, Romana Netea-Maier, Susanne I. Wudy, Christina Ludwig, Markus List, Benedikt Kirchner, Marlene Reithmair, Michael W. Pfaffl

**Affiliations:** 1 Department of Animal Physiology and Immunology, School of Life Sciences, Technical University of Munich, Freising, Germany; 2 Data Science in Systems Biology, School of Life Sciences, Technical University of Munich, Munich, Germany; 3 Department of Internal Medicine, Radboud University Medical Center, Nijmegen, The Netherlands; 4 Bavarian Center for Biomolecular Mass Spectrometry (BayBioMS), School of Life Sciences, Technical University of Munich, Freising, Germany; 5 Munich Data Science Institute, Technical University of Munich, Freising, Germany; 6 Division of Pediatric Neuro-Oncology, German Cancer Research Center, Heidelberg, Germany; 7 Institute of Human Genetics, University Hospital, Ludwig-Maximilians-University Munich, Munich, Germany

**Keywords:** Biomarker, Anaplastic thyroid cancer, Extracellular vesicles, Liquid biopsy, Drug repurposing, Transcriptomics, Biomarkers, Cancer, Computational biology and bioinformatics, Oncology

## Abstract

Anaplastic thyroid cancer (ATC) is an aggressive and rare disease. Rapid metastasis and limited treatments call for additional therapeutic options, including drug repurposing. The early spreading of ATC highlights the importance of rapid therapy success assessment, which could be achieved by measurement of extracellular vesicle (EV)-associated cell-free RNA in liquid biopsy samples. Recent studies have discovered the potential of the receptor tyrosine kinase inhibitor vandetanib for ATC treatment in vitro and in vivo. Given the rarity of ATC patients receiving off-label vandetanib treatment, acquiring patient samples for clinical studies is a prolonged process, and pre-clinical investigations are needed to elucidate the effects of vandetanib on ATC cells. Here, we present an in vitro study addressing holistic transcriptional and proteomic changes induced in the ATC cell line Cal62 by three doses of vandetanib and quantified by high-throughput methods. By comparing the transcriptional and proteomic data sets and applying dimensional reduction models such as sparse partial least-squares discriminant analysis, we refined a set of 21 biomarker candidates. Out of these, we report a final signature of eight transcriptional biomarkers, validated in cellular and cell-free RNA by RT-qPCR and verified for biological significance and discriminatory power by pathway over-representation analysis and partial least-squares regression. This transcriptional biomarker signature can distinguish vandetanib treatment from control in cell-free RNA isolated from Cal62 EVs and can be measured reliably, easily, and quickly using RT-qPCR. Our findings may serve as a basis for future clinical trials with liquid biopsy samples from ATC patients undergoing off-label vandetanib treatment.

**Supplementary Information:**

The online version contains supplementary material available at 10.1038/s41598-025-18319-w.

## Introduction

### Anaplastic thyroid cancer precision medicine

Thyroid cancer (TC) is the 8th most common type of cancer in women in the US, with a lifetime risk of 0.7% and 1.7% in men and women, respectively^1^. Accounting for less than 1% of all TC cases^2^, anaplastic thyroid cancer (ATC) is a rare but aggressive subtype, with a 5-year survival rate of less than 15% compared to an overall rate of 99% for all thyroid malignancies^1^. This low survival rate is explained by the poor differentiation of ATC and its tendency for fast growth, high inflammation and early metastasis, which is why ATC is classified as stage IV upon initial diagnosis^3,4^. Due to the usual presence of distant metastases or locally invasive extent of the disease, surgery is not a treatment option for the majority of patients, resulting in a high demand for targeted therapy options^4^. Personalized medicine may therefore provide an effective approach by analyzing each patient’s cancer-specific mutational and transcriptional profile and choosing targeted therapies tailored to those individual characteristics.

Receptor tyrosine kinases (RTKs) and their downstream signaling pathways are promising targets for such precision medicine approaches. For example, overexpression of the RTKs vascular endothelial growth factor receptor 1, 2 and 3 (VEGFR-1, -2 and -3), epidermal growth factor receptor (EGFR) and the proto-oncogene RET (“rearranged during transfection”) are commonly found in ATC^5–8^. The RTK inhibitor vandetanib specifically inhibits the activity of RET, EGFR and VEGFR-2 and -3 and is already approved in the European Union for the treatment of advanced, non-resectable medullary thyroid carcinoma (MTC), another rare subtype of TC^1,2,9^. Both the FDA and EMA approved a combination of dabrafenib and trametinib for treatment of unresectable or metastatic BRAF V600E-mutated ATC^10^. Dabrafenib inhibits the mutated BRAF kinase, while trametinib targets MEK1/2. However, this mutation is only found in 10–50% of all ATC cases, and resistance to therapy is commonly observed^3,10^. The use of NTRK inhibitors such as entrectinib is also a promising treatment option in ATC NTRK fusions and showed first successes, although those fusions are generally very rare in ATC patients^11–13^. Other promising treatment options, although not yet approved for use in ATC, include checkpoint inhibitors or PI3K inhibition (also in combination with CDK4/6 inhibitors)^14–17^. Novel emerging drugs such as the aurora kinase inhibitors alisertib or chiauranib are further promising candidates for targeted treatment; however, their full potential in ATC remains to be demonstrated^18–20^.

So far, vandetanib has not received as much attention from researchers and clinicians for off-label ATC treatment as other drugs. However, in contrast to other multi-kinase inhibitors often used off-label in ATC, such as lenvatinib, sorafenib, or pazopanib, vandetanib inhibits not only angiogenesis (VEGFR-2/3) but also proliferation (EGFR) pathways^3,21^. EGFR has been found overexpressed in a high proportion of ATC cases^22,23^. Vandetanib also showed antiproliferative and antineoplastic activity against ATC in in vitro studies with ATC cell lines and primary cell cultures from ATC biopsies, as well as in in vivo mouse experiments^6,24,25^. Vandetanib’s multitargeted inhibition of oncogenic pathways, proven clinical success in MTC, and demonstrated antitumor activity in preclinical ATC models make it a promising candidate for drug repurposing applications in ATC.

### Liquid biopsies for cancer therapy monitoring

In most research studies and clinical practice, the mutational spectrum and transcriptome of a tumor are analyzed using tissue biopsies. This invasive biopsy procedure, however, could be a burden for the patient and can be particularly challenging when metastases are located at difficult-to-reach localizations, such as the brain^2^. Tissue biopsies can also be problematic when dealing with heterogeneous tumor types like ATC, since they provide only information from a small section of the tumor^26^. As an alternative, liquid biopsies are promising diagnostic tools in tumor patients since tumor cells shed a significant amount of particles and molecules into the blood, i.e., extracellular vesicles (EVs), cell-free RNA (cfRNA), circulating tumor DNA (ctDNA) or even whole circulating tumor cells (CTCs)^27^. EVs are shed by all cells and were long thought to be only a form of waste management for cells, but in recent years, it has been shown that they can participate in several other processes, such as intercellular communication^28^. For liquid biopsy diagnostics, EVs are of particular interest since they protect associated cfRNA from RNase degradation, either by the EV lipid bilayer (for intraluminal EV-RNAs) or by steric protection through association with their protein corona^29,30^. Since the cfRNA shed by tumor cells reflects the transcriptome of the originating cell^29,31–34^, analysis of cfRNA obtained from blood samples combines information from the whole tumor, including possible distant metastases^27,35,36^. Furthermore, imaging techniques are used to monitor the cancer stages in the clinic. If the chosen therapy is not effective, this is only discovered after the tumor has had time to grow or even metastasize. Therefore, new and sensitive methods to assess a therapy’s effectiveness earlier could significantly improve the prognosis for those patients. Cell-free transcriptomic biomarkers could fill this gap, e.g., cfRNA biomarkers recently showed promising results for monitoring head and neck cancer therapy with a synergistic drug combination^37^.

**Fig. 1:**
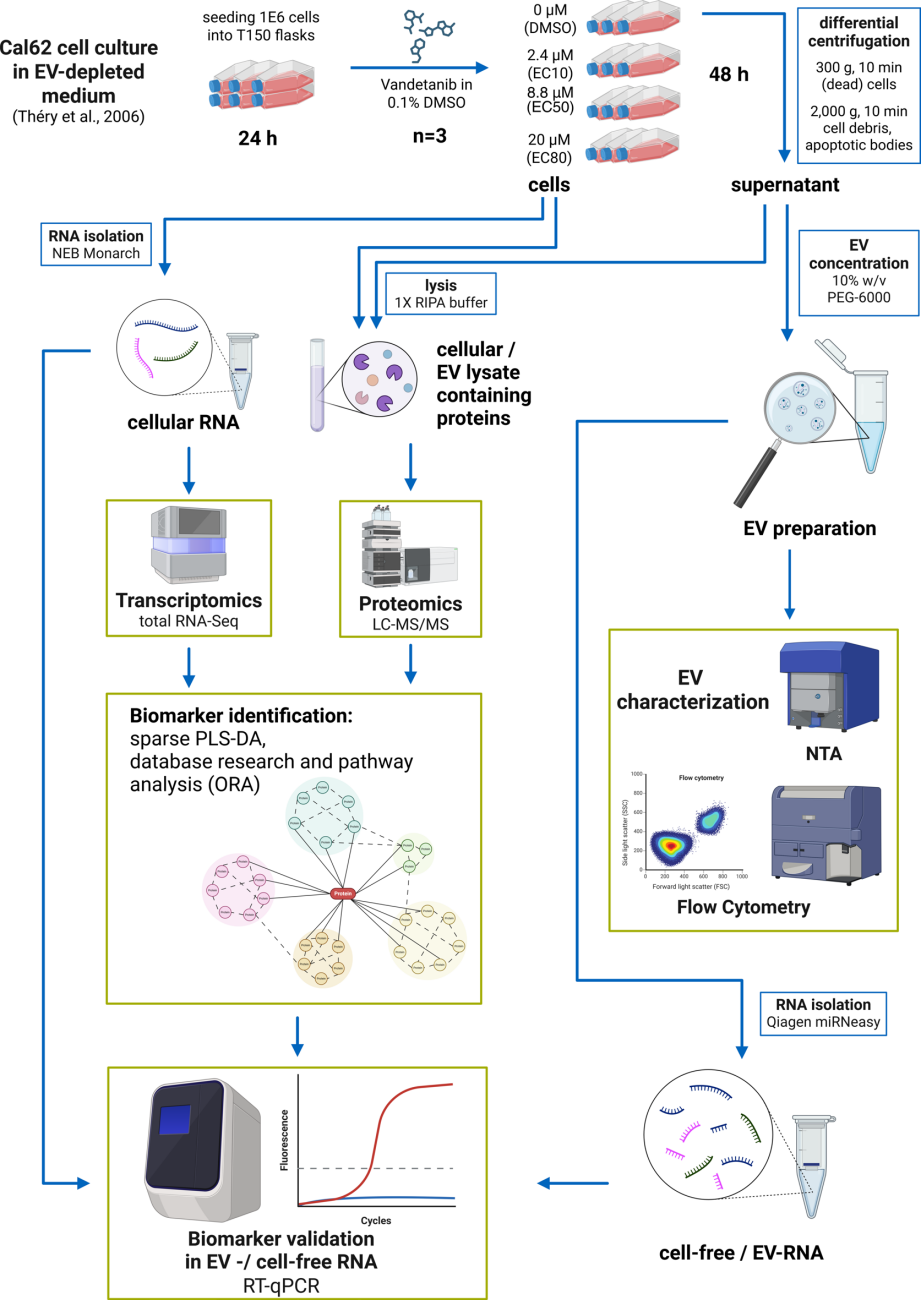
Overview of the study design, goals, and experiments. Created in BioRender.com^38^.

We aim to develop an EV-associated, cell-free transcriptomic biomarker signature for vandetanib therapy monitoring in liquid biopsy samples from ATC patients using RT-qPCR. Given the rarity of ATC and the minimal number of ATC patients receiving vandetanib, obtaining sufficient samples for comprehensive statistical analysis can take years. To address this, we utilized the ATC cell line Cal62, which has shown a promising response to vandetanib, as an in vitro model. Using total RNA sequencing (total RNA-Seq) and label-free proteomics, we investigated transcriptomic and proteomic changes in the cells induced by vandetanib. Additionally, we analyzed the EV proteome to see how the vandetanib treatment changes the secretion of proteins with EVs. Combining this information, we selected a set of biologically relevant transcriptional biomarker candidates in the cellular RNA, which we then validated in the cfRNA using reverse transcription quantitative polymerase chain reaction (RT-qPCR). Although they still require validation in plasma samples, these findings represent a critical step toward establishing a cfRNA biomarker signature for monitoring vandetanib therapy (Fig. 1).

## Results

### Study design and aims

#### Cal62 Viability

**Fig. 2:**
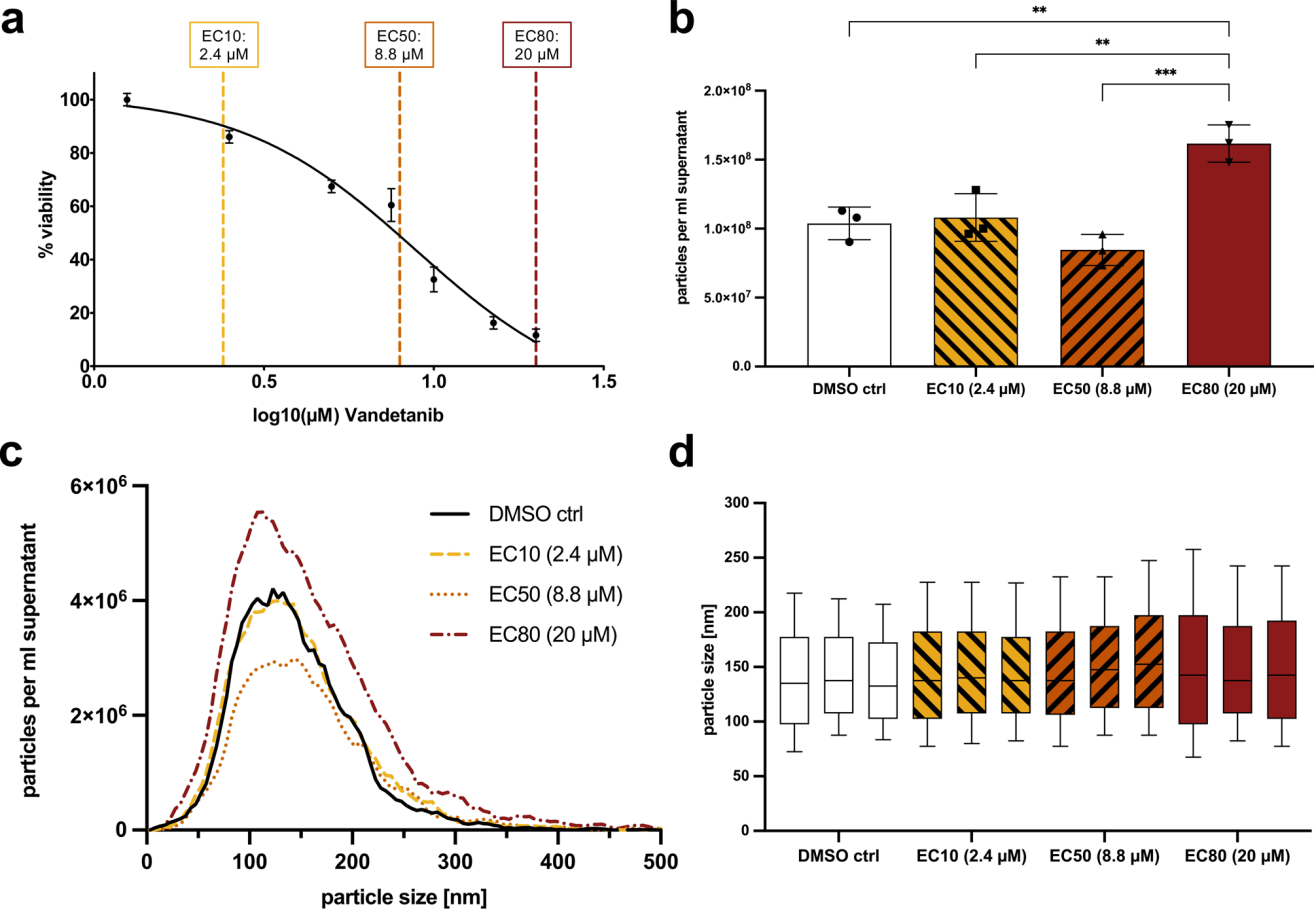
Vandetanib dose–response assessment of Cal62 cells and NTA results. (**a**) Dose–response curve of Cal62 cells treated with different doses of vandetanib for 48 h in triplicate. The EC10, EC50 and EC80 values are marked as dashed vertical lines. Error bars represent the standard error of the mean (SEM). (**b**) Mean number of particles in the EV preparations measured by NTA and normalized to the volume of cell culture supernatant. Error bars represent the SEM. One-way ANOVA with Tukey’s post-test was used to compare group means, and asterisks represent significant differences between groups with p-values < 0.01 (**) or < 0.001 (***). (**c**) Mean particle size distribution (across all three replicates) of the EV preparations measured by NTA and normalized to the volume of cell culture supernatant. (**d**) Box plots of the particle size distribution for each EV preparation measured by NTA. The lines represent the median, and the lower and upper borders represent the 25th and 75th percentiles. Whiskers represent the 10th and 90th percentiles. One-way ANOVA with Tukey’s post-test was used to compare group medians, and no significant differences were found.

Cal62 cells showed sensitivity to vandetanib treatment for 48 h in the low micromolar range, as shown in the dose-response curve in (Fig. 2a). The EC10, EC50 and EC80 values were determined as 2.4, 8.8 and 20 µM, respectively. Besides Cal62, we also evaluated the ATC cell line 8505C in a preliminary experiment. 8505C also shows high expression levels of EGFR and harbors the mutations BRAF V600E and TP53 R248, among others^39,40^. Because Cal62 cells proved to be slightly more susceptible to vandetanib (Supplementary Fig. S1), we decided to use Cal62 in this study.

#### EV Characterization

**Fig. 3:**
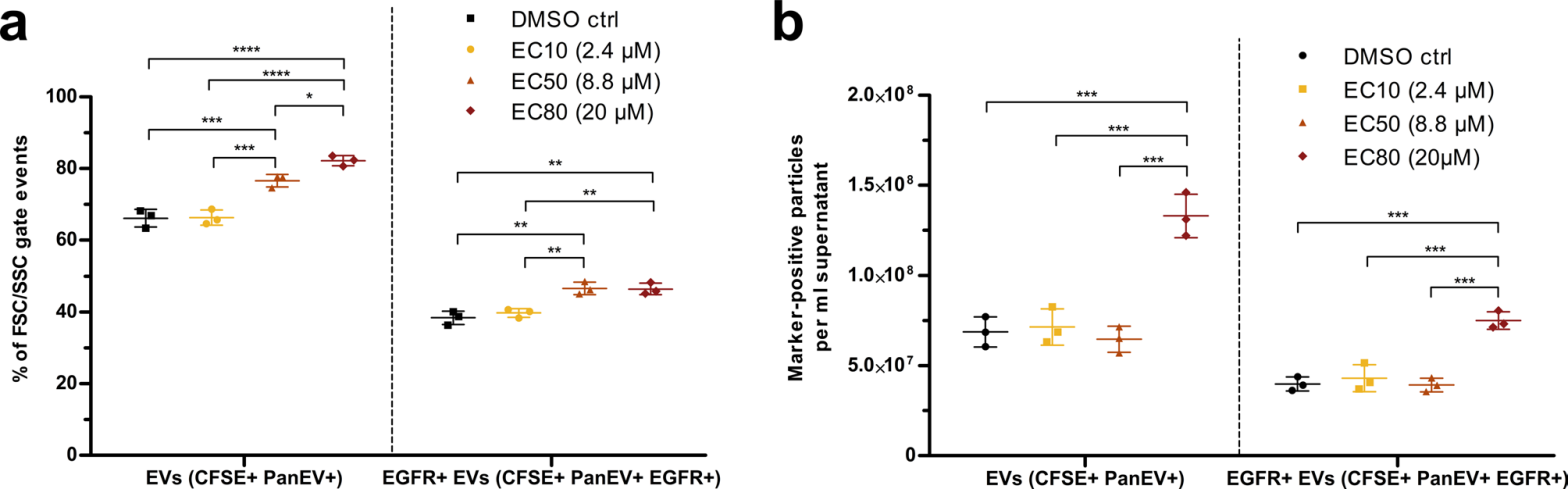
EV flow cytometry results. Group means are shown as lines, and whiskers represent the SEM. One-way ANOVA was used to compare the different treatments. Asterisks represent significant changes with p-values < 0.05 (*), < 0.01 (**), < 0.001 (***) and < 0.0001 (****). (**a**) Percentages of positive events for CFSE and PanEV staining (EVs, left) and additionally for EGFR staining (EGFR + EVs, right). (**b**) Number of particles that were positive for CFSE and PanEV staining (EVs, left) and additionally for EGFR staining (EGFR + EVs, right), normalized to the volume of cell culture supernatant used for EV concentration.

Nanoparticle tracking analysis (NTA) showed a significant increase in particles per ml cell culture supernatant upon treatment with the EC80 dose (20 µM) of vandetanib compared to the DMSO control and the lower vandetanib doses (Fig. 2b,c). One-way ANOVA with Tukey’s post-test found no significant differences between group particle size medians (Fig. 2d). Flow cytometry (FC) analysis revealed that 63–84% of size-gated events were positive for CFSE and the tetraspanin EV markers CD9, CD63, and CD81 (PanEV), and were therefore classified as EVs. 36%-48% of events stained additionally positive for EGFR (EGFR^+^ EVs, Fig. 3a). The proportions of both total EVs and EGFR⁺ EVs increased significantly following treatment with the EC50 and EC80 doses of vandetanib, compared to DMSO and EC10. Notably, the EC80 dose also resulted in a significantly higher EV proportion than EC50. Normalized to cell culture supernatant volume, 6.1E7 – 1.5E8 EVs/ml and 3.6E7 – 8.1E7 EGFR^+^ EVs/ml were quantified by FC. The absolute number of (EGFR^+^) EVs/ml supernatant was significantly increased in the EC80 dose compared to DMSO, EC10 and EC50, whereas no increase was observed in the EC50 dose relative to the control or EC10. RNA integrity numbers (RIN) calculated on the BioAnalyzer were between 2.4 and 3.3 for all EV RNA samples.

#### Transcriptomics

**Fig. 4:**
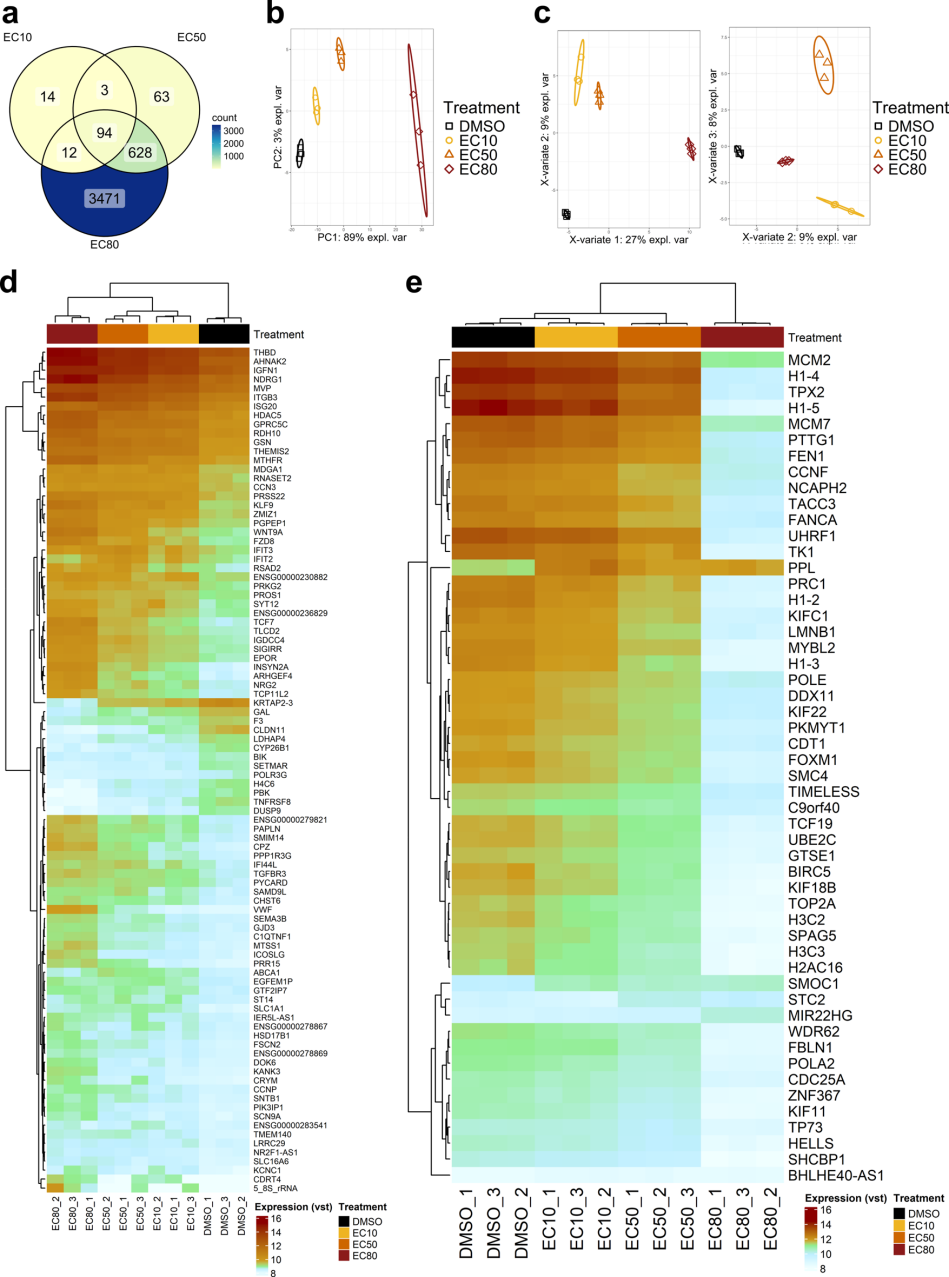
Cellular transcriptomics results. (**a**) Venn diagram of differentially expressed genes identified by RNA-Seq in the different vandetanib doses compared to DMSO. (**b**) Two-dimensional reduction of the 500 genes with the highest variance in the variance-stabilized RNA-Seq dataset by principal component analysis (PCA). Black squares, yellow dots, orange triangles and red diamonds indicate the DMSO control, EC10, EC50 and EC80 dose, respectively. Ellipses represent 95% confidence intervals (CIs). (**c**) Three-dimensional reduction of the variance-stabilized RNA-Seq data by sparse partial least-squares discriminant analysis (sPLS-DA). Tuning of the model resulted in choosing three components with 50, 40 and 25 factors each. The plots show the samples in the first and second (left) and in the second and third (right) dimensions. Black squares, yellow dots, orange triangles and red diamonds indicate the DMSO control, EC10, EC50 and EC80 dose, respectively. Ellipses represent 95% CIs. (**d**) Heatmap representing the variance-stabilized (vst) expression data of the 94 genes identified as differentially expressed in all three vandetanib treatments compared to DMSO. (**e**) Heatmap representing the variance-stabilized (vst) expression data of the 52 genes that were picked by the sPLS-DA model and significantly differentially expressed in at least one vandetanib dose compared to the DMSO control.

Cellular transcriptomics showed that treatment of Cal62 cells with vandetanib had a large effect on gene expression. In the cellular RNA, total RNA-Seq revealed significant differential expression (padj < 0.05 and absolute log2 fold change (log2FC) ≥ 1) of 4,283 genes upon vandetanib treatment compared to the DMSO control, 2,177 of which were upregulated and 2,109 downregulated (Supplementary Table S1). Treatment with the EC10 / EC50 / EC80 dose of vandetanib resulted in differential expression of 123 / 792 / 4209 genes compared to the DMSO control, respectively (Fig. 4a and Supplementary Fig. S2).

Dimensional reduction of the variance-stabilized dataset using principal component analysis (PCA) of the top 500 genes with the highest variance, separated the samples dose-dependently correlating to the vandetanib treatment in the first dimension, which also explained 89% of the variance in the data (Fig. 4b). For biomarker candidate selection, we pre-selected the 94 genes found differentially expressed in all doses (Fig. 4a,d). Furthermore, we performed sparse partial least-squares (sPLS) regression analysis (3 components, 50/40/25 factors, respectively) to identify the genes that contributed most to the separation of the treatment groups (Fig. 4c,e). The 52 differentially expressed genes picked by the sPLS model were also included in the biomarker selection process.

To further evaluate the specificity of our biomarker signature for vandetanib, we compared our results with publicly available drug perturbation data from the LINCS project^41^. Specifically, we selected three compounds – doxorubicin (LINCS accession BRD-K92093830), mitomycin C (BRD-A48237631), and actinomycin D (BRD-A73909368) – which are known to exert broad cytotoxic effects independent of vandetanib’s mechanism of action. Analysis of the transcriptional responses induced by these agents in the LINCS-tested cell lines revealed that the expression of our eight biomarker candidates was not consistently altered in the same direction as observed in vandetanib-treated Cal62 cells. Spearman correlation analyses between our RNA-Seq log2FC values at all three vandetanib doses and the mean LINCS Z-scores (10 µM, 24 h, across all cell lines) generally revealed very low correlations (Table 1). The only significant correlation was observed between mitomycin C and the EC50 vandetanib dose, with a Spearman’s rho of 0.83 and a p-value of 0.0154. All eight other comparisons showed no significant correlation (p-value < 0.05 and rho value > 0.6).

**Table 1:** Spearman correlation between vandetanib-induced log2FC in Cal62 (at all three tested doses) and mean LINCS Z-scores (10 µM, 24 h) across all cell lines for the eight biomarker candidates.

Comparison	Spearman ‘s rho	p-value
Actinomycin D vs vandetanib EC10	-0.19	0.6646
Actinomycin D vs vandetanib EC50	0.29	0.5008
Actinomycin D vs vandetanib EC80	0.14	0.7520
Doxorubicin vs vandetanib EC10	0.07	0.8820
Doxorubicin vs vandetanib EC50	0.43	0.2992
Doxorubicin vs vandetanib EC80	0.29	0.5008
Mitomycin C vs vandetanib EC10	0.55	0.1710
Mitomycin C vs vandetanib EC50	0.83	0.0154
Mitomycin C vs vandetanib EC80	0.69	0.0694

#### Proteomics

**Fig. 5:**
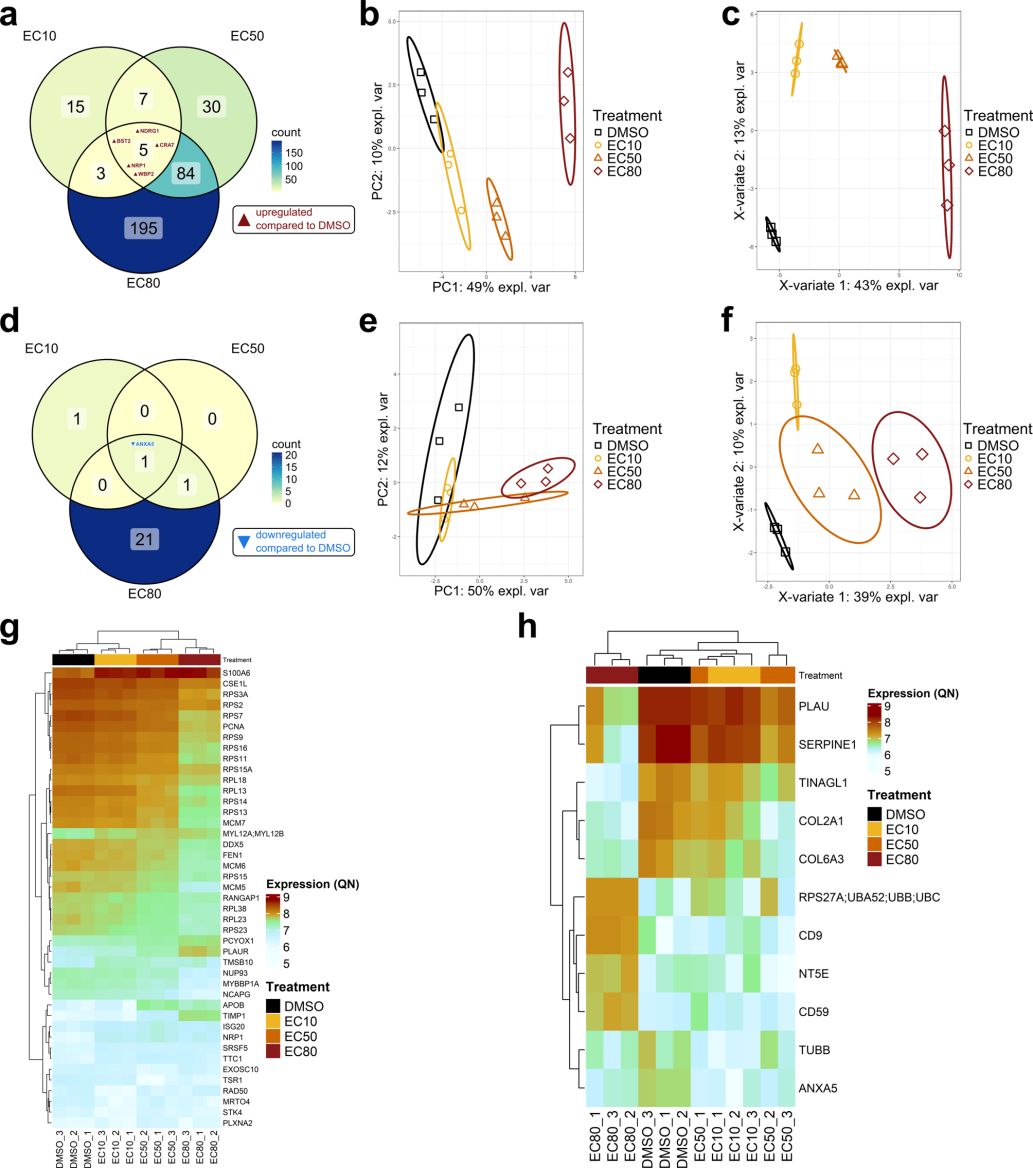
Cellular and EV proteomics results. a + d. Venn diagrams of differentially expressed cellular (**a**) and EV (**d**) proteins identified by label-free proteomics in the different vandetanib doses compared to DMSO. The gene names of the differentially expressed proteins in all vandetanib treatments compared to DMSO were added to the diagrams. b + e. Two-dimensional reduction of the 500 proteins with the highest variance in the quantile-normalized proteomics dataset by principal component analysis (PCA) in the cellular (**b**) and EV (**e**) proteome. Black squares, yellow dots, orange triangles and red diamonds indicate the DMSO control, EC10, EC50, and EC80 dose, respectively. Ellipses represent 95% CIs. c + f. Three-dimensional reduction of the quantile-normalized cellular (**c**) and EV (**f**) proteomics data by sparse partial least-squares discriminant analysis (sPLS-DA). Tuning of the model resulted in choosing three components with 47, 41 and 45 factors each (10, 3 and 24 for EVs). The plots show the samples in the first two dimensions. Black squares, yellow dots, orange triangles and red diamonds indicate the DMSO control, EC10, EC50 and EC80 dose, respectively. Ellipses represent 95% CIs. g + h. Heatmap representing the quantile-normalized (QN) expression data of the 43 (**g**) / 11 (**h**) proteins that were picked by the sPLS-DA model and significantly differentially expressed in at least one vandetanib dose compared to the DMSO control on the cellular (**g**) / EV (**h**) level.

A total of 2,325 proteins were detected in the cellular samples via label-free proteomics, with 441 significantly (padj < 0.05 and absolute log2FC ≥ 1) altered upon vandetanib treatment – 236 up- and 205 downregulated (Supplementary Table S2). In EVs, 68 proteins were detected, 29 of which were differentially expressed: 15 up- and 14 downregulated (Supplementary Table S3). Five cellular proteins (BST2, CRAT, NDRG1, NRP1, and WBP2) were consistently upregulated across all three vandetanib doses compared to DMSO (Fig. 5a). In contrast, only ANXA5 was consistently downregulated in EVs across all doses, with most differentially expressed EV proteins (21 of 24) detected exclusively at the EC80 dose (Fig. 5d). As in the transcriptomics data, PCA of the quantile-normalized protein expression values (top 500 proteins by variance) showed dose-dependent separation, more pronounced in cellular samples (Fig. 5b) than in EVs (Fig. 5e). A three-component sPLS-DA model of the cellular data (47/41/45 factors) identified 43 differentially expressed proteins (Fig. 5c,g). Similarly, the EV sPLS-DA model (10/3/24 factors) identified 11 differentially expressed proteins (Fig. 5f, h).

**Fig. 6:**
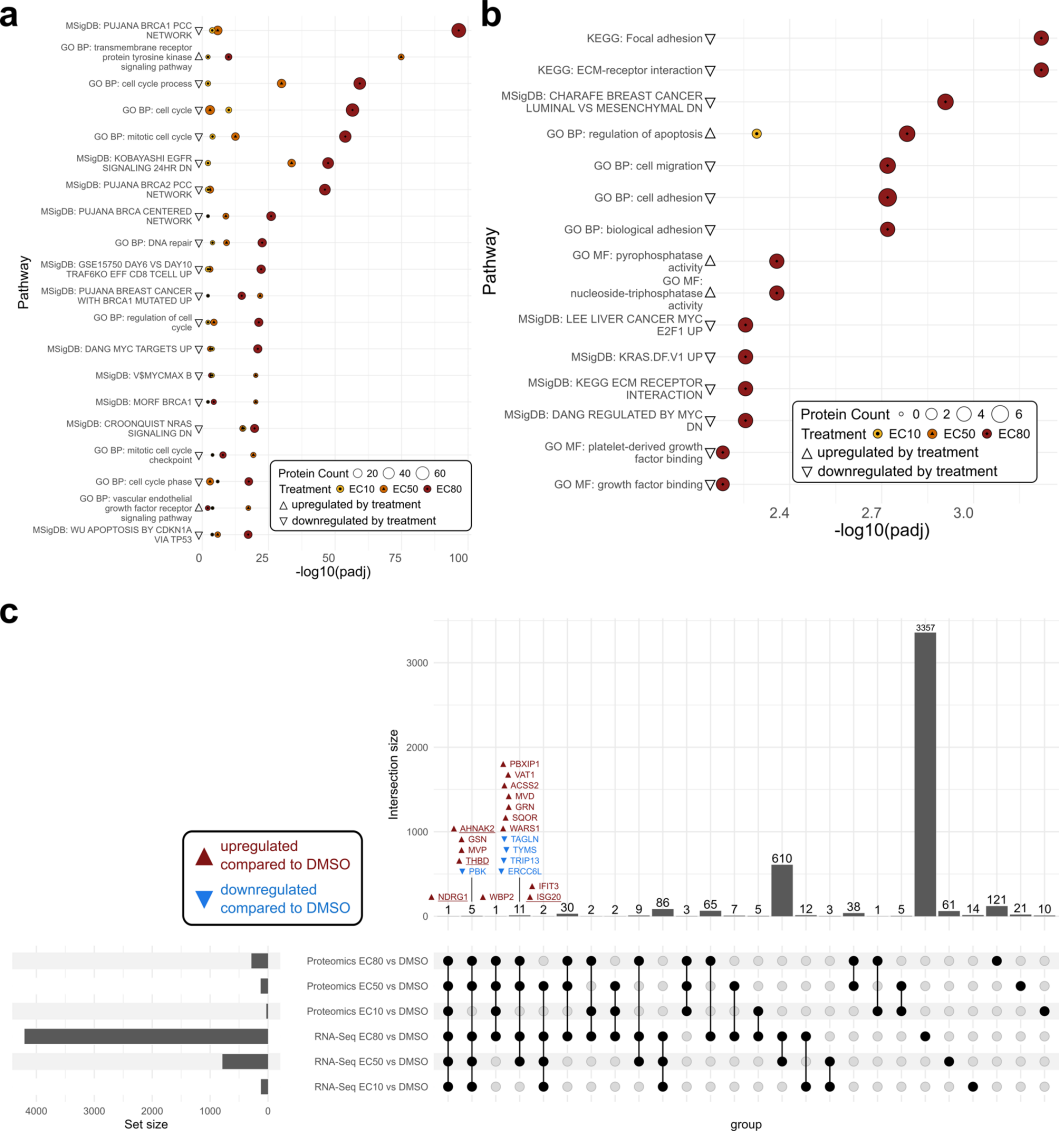
Proteomic pathway ORA results and comparison of the transcriptomic and proteomic data sets. (**a**) Top 20 pathways found enriched by ORA in the cellular proteome, filtered for cancer-related pathways and sorted according to the smallest padj value. (**b**) Cancer-related pathways found enriched by ORA in the EV proteome, sorted according to the smallest padj value. a + b. The y-axis shows the respective database as well as the name of the pathway. The circle diameter shows the number of proteins participating in the pathway that were found differentially expressed in the proteomics data. Colors and symbols indicate the treatment in which the pathway was found to be significantly enriched compared to the DMSO control. GO BP = Gene Ontology Biological Process; GO MF = Gene Ontology Molecular Function; MSigDB: Molecular Signatures Database; KEGG: Kyoto Encyclopedia of Genes and Genomes^42–44^. (**c**) UpSet plot of the genes found differentially expressed on both cellular transcriptomic and proteomic levels. The gene names found differentially expressed in at least four different treatment-analysis combinations were added to the plot. Underlined genes are part of the final biomarker signature proposed below.

Pathway over-representation analysis (ORA) of the cellular proteomics data revealed 1,087 significantly regulated pathways (padj < 0.05) across all three vandetanib doses compared to DMSO, with 727 associated with upregulated and 360 with downregulated proteins (Supplementary Table S4). Out of these, 68 were cancer-related (38 upregulated, 30 downregulated). The top 20 of these (by smallest padj) are shown in Fig. 6a, and together with the remaining 48 in Supplementary Figure S3. In the EV proteome, ORA identified 194 significantly regulated pathways (padj < 0.05), although none were consistently altered across all doses. Specifically, eight pathways were regulated at EC10 (3 up, 5 down), 19 at EC50 (1 up, 18 down), and 181 at EC80 (71 up, 110 down). Three pathways were shared between EC10 and EC80, and six between EC50 and EC80 (Supplementary Table S4). Among the 194 pathways, 15 were cancer-related (3 associated with upregulated and 12 with downregulated proteins), as shown in (Fig. 6b).

Comparison of cellular RNA-Seq and proteomics data revealed that *NDRG1* was consistently upregulated across all vandetanib doses at both RNA and protein levels. Additionally, four genes (*AHNAK2*, *GSN*, *MVP*, and *THBD*) were upregulated, and *PBK* was downregulated in all treatments on the transcriptional level and in the two highest doses (EC50 and EC80) on the protein level. *ISG20* and *IFIT3* were upregulated at all vandetanib doses in the RNA-Seq but only at the EC50 dose in the proteomics data. *WBP2* was upregulated in all three doses on the protein level but only at the highest dose (EC80) on the RNA level. Additionally, seven genes (*PBIXP1*, *VAT1*, *ACSS2*, *MVD*, *GRN*, *SQOR*, and *WARS1*) were upregulated, and four (*TAGLN*, *TYMS*, *TRIP13*, and *ERCC6L*) downregulated at both RNA and protein levels in the EC50 and EC80 treatments. These findings are summarized in the UpSet Plot (Fig. 6c), and the highlighted genes were further considered as additional potential biomarker candidates.

Comparison of cellular RNA-Seq and proteomics data revealed that *NDRG1* was consistently upregulated across all vandetanib doses at both RNA and protein levels. Additionally, four genes (*AHNAK2*, *GSN*, *MVP*, and *THBD*) were upregulated, and *PBK* was downregulated in all treatments on the transcriptional level and in the two highest doses (EC50 and EC80) on the protein level. *ISG20* and *IFIT3* were upregulated at all vandetanib doses in the RNA-Seq but only at the EC50 dose in the proteomics data. *WBP2* was upregulated in all three doses on the protein level but only at the highest dose (EC80) on the RNA level. Additionally, seven genes (*PBIXP1*, *VAT1*, *ACSS2*, *MVD*, *GRN*, *SQOR*, and *WARS1*) were upregulated, and four (*TAGLN*, *TYMS*, *TRIP13*, and *ERCC6L*) downregulated at both RNA and protein levels in the EC50 and EC80 treatments. These findings are summarized in the UpSet Plot (C), and the highlighted genes were further considered as additional potential biomarker candidates.

#### Biomarker validation

To refine the initial set of 158 biomarker candidates, genes with a baseMean < 700 in the DESeq2-analyzed cellular RNA-Seq data were excluded, as preliminary experiments showed that low-expression genes were not detectable by RT-qPCR in the cfRNA. The remaining candidates were further filtered based on biological relevance, retaining only genes with established roles in cancer biology through literature and database research. This resulted in a refined set of 21 transcriptional biomarker candidates (Table 2). Pathway ORA of these 21 genes using all genes detected in the RNA-Seq experiment as background found 42 pathways significantly enriched (padj < 0.1), the majority of which are involved in key processes related to cancer progression, including proliferation, growth, and cell cycle regulation (Fig. 8a). Eight of the 21 candidates – *NDRG1, FOXM1, TPX2, ISG20, MYBL2, AHNAK2, GPRC5C,* and *THBD –* could be validated by RT-qPCR in cellular RNA, and the first five of them also in EV-associated cfRNA. The last three of the eight genes were detected by RT-qPCR in cfRNA, but showed inverse regulation compared to the cellular RNA. For the other 13 biomarker candidates, validation failed in the cfRNA due to low expression levels. Across all eight genes, cellular RT-qPCR log2FC values closely matched those from DESeq2 analysis.

**Table 2:** The refined set of 21 transcriptional biomarker candidate genes.

*AHNAK2*	*ISG20*	*NCAPD2*
*BIRC5*	*ITGB3*	*NDRG1*
*CDC25A*	*KLF9*	*RDH10*
*FOXM1*	*LAMC3*	*THBD*
*GPRC5C*	*MTHFR*	*TOP2A*
*GSN*	*MVP*	*TPX2*
*HDAC5*	*MYBL2*	*WNT9A*

**Fig. 7:**
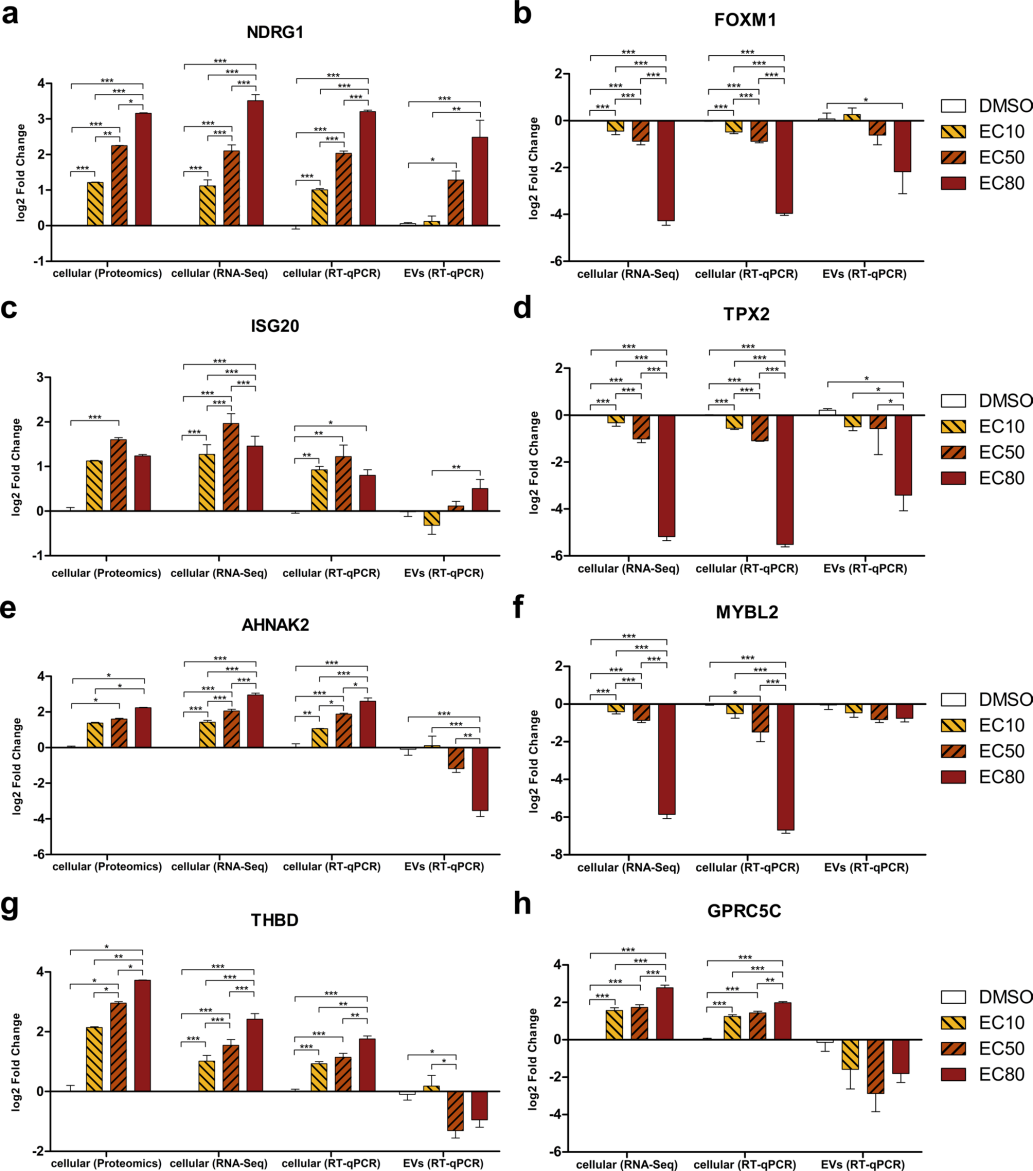
Expression values of the eight validated biomarkers on the protein and transcriptional level. Bar charts show the mean log2FC on protein and cellular and cell-free RNA level measured by label-free proteomics (left, **a**, **c**, **e**, **g** only) total RNA-Seq (second from left in **a**, **c**, **e**, **g** and left in **b**, **d**, **f**, **h**, cellular RNA only) and RT-qPCR (second from right: cellular RNA, right: cfRNA). Error bars represent the SEM, and asterisks indicate significant changes with p-values < 0.05 (*), < 0.01 (**) and < 0.001 (***) as assessed by Student’s t-test with Benjamini–Hochberg multiple testing correction (proteomics) and one-way ANOVA (RNA-Seq and RT-qPCR). For ANOVA, Dunnett’s post-test was used to compare treatment with the DMSO control, while the different vandetanib concentrations were compared using Tukey’s post-test. Each diagram shows the data for a different biomarker candidate gene: NDRG1 (**a**), FOXM1 (**b**), ISG20 (**c**), TPX2 (**d**), AHNAK2 (**e**), MYBL2 (**f**), THBD (**g**), and GPRC5C (**h**). FOXM1, TPX2, MYBL2, and GPRC5C were not detected on protein level.

Among the eight validated biomarker candidates (Fig. 7), *NDRG1* was significantly upregulated in the EC50 and EC80 in the cfRNA, closely matching the upregulation at the cellular RNA and protein level. *FOXM1* and *TPX2* showed significant downregulation at the EC80 dose in the cfRNA, consistent with the cellular RNA results. *ISG20* was significantly upregulated in the EC80 dose relative to EC10 in the cfRNA, again in agreement with the cellular RNA and protein levels. For *MYBL2*, although no significant regulation was detected in the cfRNA, the expression trend was consistent with the downregulation seen in the cellular RNA. The remaining three genes – *AHNAK2*, *GPRC5C*, and *THBD* – were inversely regulated, being upregulated in the cellular RNA but downregulated in cfRNA. *AHNAK2* was significantly downregulated in the cfRNA at EC80 and *GPRC5C* showed consistent downregulation across all vandetanib doses in cfRNA compared to DMSO, although this did not reach statistical significance. *THBD* was significantly downregulated in cfRNA at the EC50 dose. For *AHNAK2* and *THBD*, the regulation at the cellular RNA level was close to that detected at the protein level. *FOXM1*, *TPX2*, *MYBL2*, and *GPRC5C* were not detected at the protein level. Mean log2FCs and p-values for all genes and treatments are listed in Supplementary Table S5.

**Fig. 8:**
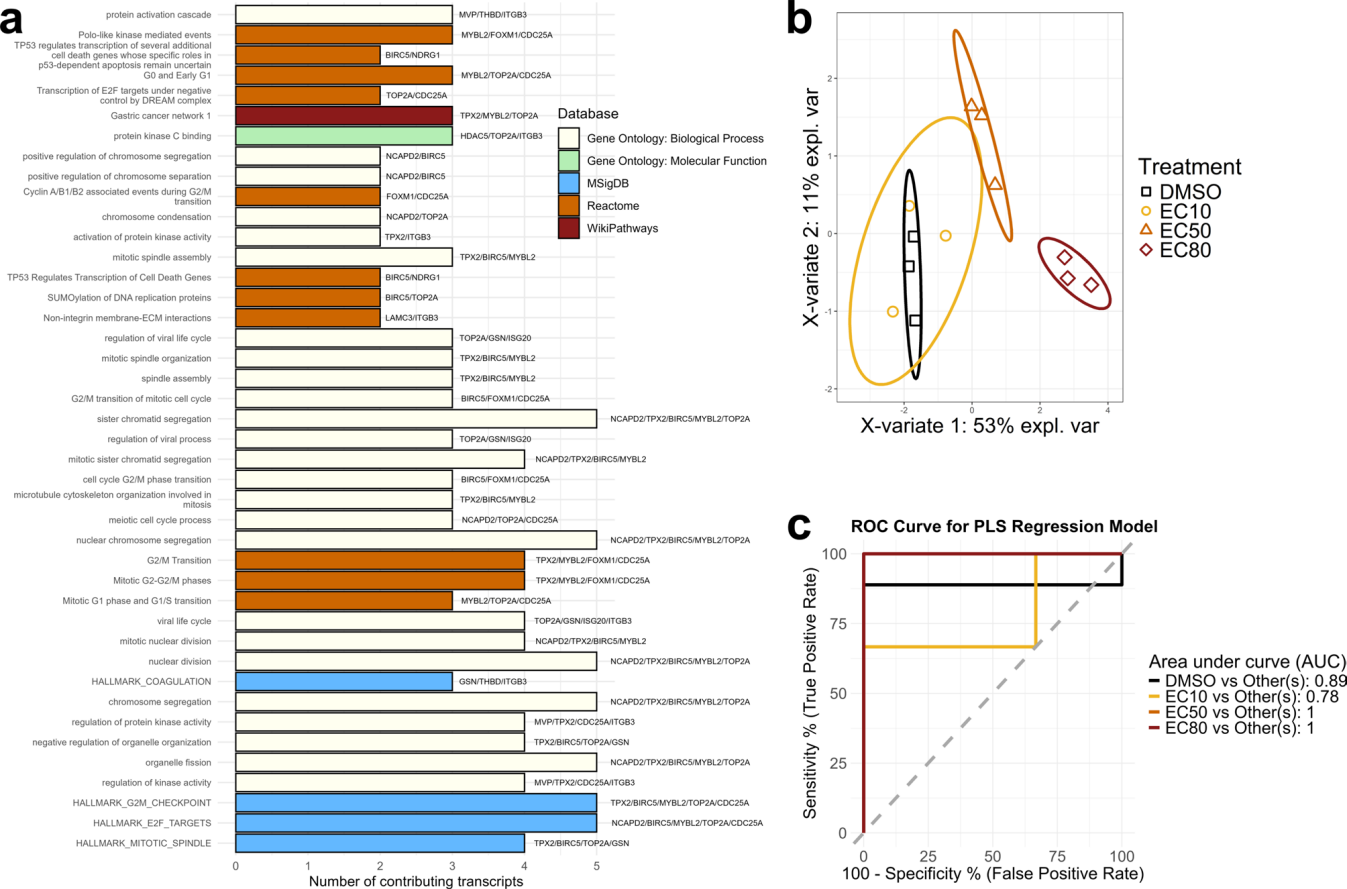
Biological relevance and discriminatory power of the biomarker candidates. (**a**) Pathway ORA of the 21 biomarker candidates in the total RNA-Seq dataset, filtered by padj < 0.1 and sorted by fold enrichment. Pathway names are indicated on the y-axis, and colors represent the corresponding database. The names of the biomarker candidate genes contributing to each pathway are indicated next to the bars. (**b**) Two-dimensional projection of the samples in the first two dimensions of a partial least-squares regression of the delta Cq values measured in the cfRNA. Black squares, yellow dots, orange triangles and red diamonds indicate the DMSO control, EC10, EC50 and EC80 dose, respectively. Ellipses represent 95% CIs. (**c**) Receiver operating characteristic (ROC) curves and area under the curve (AUC) values for the PLS regression model of the eight biomarker candidates’ delta Cq values.

The discriminatory potential of the validated biomarkers in the cfRNA was then assessed using a PLS regression model with two components, based on RT-qPCR delta-Cq values. This model successfully separated the EC50 and EC80 dose treatment of vandetanib from the DMSO control by the RT-qPCR delta-Cq values (Fig. 8b). Receiver-operating characteristic (ROC) analysis further confirmed the model’s high discriminatory power (Fig. 8c), achieving perfect classification of the EC80 and EC50 treatment from the other treatments with an area under the curve (AUC) of 1.00, strong discrimination of DMSO from the vandetanib treatments (AUC = 0.89) and acceptable separation of the EC10 treatment from other conditions (AUC = 0.78).

## Discussion

We have demonstrated that vandetanib induces severe expression changes in the transcriptome and proteome of ATC cells in all tested doses and report a signature of eight EV-associated genes, which indicate the anti-proliferative effect of vandetanib on the cells. Out of the 21 biomarker candidates, we successfully validated eight in the cellular RNA, meaning that the log2FCs assessed by RT-qPCR were very close to the results calculated with *DESeq2* from the RNA-Seq experiment. Additionally, the log2FCs of the eight biomarkers that could be detected at the cellular protein level by label-free proteomics were very close to those measured in the cellular RNA. Since the biomarker signature should ultimately be measured by RT-qPCR in cell-free RNA to enable its potential use for therapy monitoring in liquid biopsy samples, the validation experiment was performed not only in the cellular but also in the EV-RNA. For five genes, the direction of regulation was identical to the cellular level. Interestingly, the regulation of *AHNAK2*, *GPRC5C*, and *THBD* in the EV-RNA was inverse to that measured in the cellular RNA. For *AHNAK2* and *THBD* this inverse effect was significant, while for *GPRC5C* no significance was detected, although the results showed strong downregulation in all three vandetanib doses. These findings indicate that although more mRNA is produced for these three genes in the cells upon vandetanib treatment, the RNA might selectively *not* be sorted into EVs. While miRNA sorting to EVs is slowly being unraveled, sorting of mRNA is still a relatively unexplored topic, but there is evidence that RNA-binding proteins are one mechanism for targeting specific sequences into EVs^45^. Similar inverse mRNA regulation in cells and their EVs has been reported in 2022 by O’Grady et al*.*^46^. After stimulating human umbilical vein endothelial cells with VEGF, they observed downregulation of mRNAs in EVs that were upregulated in the cells and vice versa. Additional experiments led them to propose that cells can selectively sort RNA to EVs as a mechanism to regulate cellular expression levels. Our results indicate that the same could be true for *AHNAK2*, *GPRC5C* and *THBD*, with the vandetanib treatment increasing the amount of mRNA in the cells by downregulating its sorting to EVs. Further experiments would be needed to verify this hypothesis and further assess the underlying mechanism of the observed inverse regulation, which is beyond the scope of this paper. The other five biomarker candidates showed the same direction of regulation in the cell-free as in the cellular RNA.

Considering these results, we ultimately propose a signature of eight transcriptional biomarkers for assessing the vandetanib treatment of Cal62 cells in vitro in the EV-associated cfRNA: Upregulation of *NDRG1* and *ISG20* with simultaneous downregulation of *FOXM1*, *TPX2*, *MYBL2*, *AHNAK2*, *GPRC5C* and *THBD*. The expression of these genes can be assessed easily, quickly, and cost-efficiently by RT-qPCR, for instance, using the primers and reference genes described above. For expression measurements of these genes in cellular samples, we want to point out the caveat that *AHNAK2*, *GPRC5C* and *THBD* are inversely regulated in the cells compared to the EVs, as explained above.

ORA of the eight biomarker candidates highlighted their biological significance in the context of ATC and RTK inhibition, since all significantly enriched pathways were related to cancer-related processes like kinase signaling or cell cycle regulation. Furthermore, the PLS model generated with the cell-free RT-qPCR data could clearly separate the EC50 and EC80 doses of vandetanib from the DMSO and EC10 treatments, indicating a good discriminatory power of the proposed biomarker signature. However, the EC10 treatment could not be separated from the DMSO control since the FC induced by vandetanib in that dose in the cfRNA was very small for most of the biomarkers. These results, together with the ROC AUC values, confirmed that the eight biomarkers were able to differentiate between the EC50 and EC80 doses and all other treatments, and discriminate the DMSO treatment well from all vandetanib treatments.

The EC50 value of 8.8 µM is consistent with previously reported values for vandetanib in thyroid cancer cell lines. For example, Walter et al*.* observed an EC50 of 5.0 µM in TT cells, a medullary thyroid carcinoma cell line that harbors the activating RET Mutation C634W, and Ferrari et al*.* reported a range of 4.7 – 13.0 µM in several different ATC cell lines^24,47^. Notably, much lower effective concentrations of vandetanib have been described in TT cells, e.g., by Vitagliano et al*.* (50–500 nM)^48^. However, those values were obtained using different, more sensitive assays. Whereas the present study measured metabolic activity using an MTT-based assay, Vitagliano et al*.* employed a bromodeoxyuridine (BrdU) incorporation assay, which detects DNA synthesis. The methodological differences likely account for the various concentrations reported in the different publications. FC confirmed that most of the detected particles were indeed EVs and a big proportion also showed high expression of the vandetanib target EGFR. The number and percentage of (EGFR^+^) EVs produced by Cal62 cells significantly increased through vandetanib treatment, likely due to increased cellular stress, since it is well known that different forms of stress can increase EV secretion^49^. Interestingly, the proportion of (EGFR^+^) EVs was elevated at both the EC50 and EC80 doses, whereas a significant increase in the absolute number per ml supernatant was observed only at the highest dose. Although additional studies are required to elucidate the underlying mechanisms, a plausible explanation is that a reduction of non-EV particles at both EC50 and EC80 doses led to an increased relative abundance of EVs and EGFR^+^ EVs within the total particle population. This interpretation is supported by the NTA results, which showed a slight decrease in total particle concentration at the EC80 dose, possibly reflecting diminished non-EV particle levels and consequently an increased percentage of EVs. Only the EC80 dose of vandetanib resulted in increased production of EVs, which likely explains why the number of (EGFR^+^) EVs per ml supernatant was elevated exclusively at this concentration.

Besides the reduced cellular viability, this increased cellular stress, causing elevated release of EVs, is another indicator that the vandetanib treatment impaired the proliferation of Cal62 cells. Since the median particle size did not differ between groups, it is unlikely that the increased particle number was caused by apoptotic bodies produced from dying cells, as those particles are generally much larger than exosomes and ectosomes. Given those results, we demonstrated that precipitation yields particles predominantly positive for EV markers, despite being a crude method^50^. Compared to other commonly used EV isolation or concentration techniques, precipitation offers high cfRNA recovery while remaining inexpensive, rapid, and easy to implement^51–54^. It requires only a standard swinging-bucket centrifuge – equipment available in most diagnostic laboratories, making it highly compatible with clinical workflows. Since the reproducibility of cfRNA biomarker studies is inherently dependent on the protocols used for sample collection, EV isolation, RNA extraction, and analysis, it is important that the discovery workflow closely mirrors the methods intended for later clinical implementation^54–57^. This ensures the reliability, validity, and eventual translational potential of the identified biomarkers.

Dimensional reduction by PCA confirmed that the transcriptional and proteomic changes induced by vandetanib treatment were the predominant effect in the data. When looking for treatment response biomarker candidates, genes that are differentially expressed throughout a broad range of treatment concentrations are of special interest. In this study, 94 genes were differentially expressed in all vandetanib doses compared to DMSO. Additionally, potential biomarkers should enable clear discrimination between the treatment and control, such as the 52 genes picked by the sPLS-DA model^57^.

The five cellular proteins found differentially expressed in all treatments were all upregulated but not associated with a common function or pathway. Notably, the protein N-Myc Downstream Regulated 1 (NDRG1) is involved in the recycling and sorting of endosomes, and specifically the recycling of E-Cadherin, indicating that it might be a suppressor of metastasis^58^. Transcription of *NDRG1* is regulated by the N-Myc transcription factor, which is activated by the MEK/ERK signaling cascade downstream of RTK signaling^59^. By inhibiting RTK signaling, vandetanib therefore most likely induces the differential expression of *NDRG1* through the MEK/ERK pathway. Another upregulated protein, Neuropilin 1 (NRP1), has been characterized as a coreceptor for VEGF, enhancing the binding of a specific VEGF isoform to VEGFR-2^60^. Upregulation of NRP1 might therefore be an attempted escape mechanism as a consequence of the VEGF-signaling inhibition induced by vandetanib. The next protein, WBP2, was identified a few years ago as an oncogenic transcription factor connected to several important pathways involved in tumorigenesis, including EGFR signaling^60^. Tetherin (BST2) is an important protein for antiviral defense, but has also been shown to initiate cell cycle progression and therefore overcome EGFR inhibition in oral squamous cell carcinoma^61^. Finally, carnitine O-acetyltransferase (CRAT) has also been associated with several tumors and has, in mice, been linked to cell cycle progression^62^.

Comparison of the RNA-Seq and proteomics data showed that NDRG1 was upregulated on both RNA and protein levels in all doses. This means that not only was the RNA, which would ultimately be the analyte of a PCR-based biomarker test, regulated in all tested treatments, but also that the biologically active protein was affected in the cells in all tested vandetanib doses. This result underlined the suitability of NDRG1 as a possible biomarker, as it was also identified by multiple other analyses described above.

Interestingly, only Annexin V (ANXA5) was downregulated in all vandetanib treatments in the EVs. Although more popular for its capacity for binding to phosphatidylserine in the EV membrane and commonly used to stain EVs for fluorescence analysis, Annexin V and other Annexins are also regularly found as part of the cytosolic cargo of EVs and were suggested as protein markers for EV characterization in the 2018 MISEV guidelines^63^. Additionally, Annexin V has been reported to interact with VEGFR-2, the most important target of vandetanib^64^. Unfortunately, there seems to be no further research analyzing this interaction, although other Annexins have also been associated with VEGFR-2 signaling, e.g., Annexin A8^65^. On the protein level, VEGFR-2 was not detected, but the transcriptomics data showed that VEGFR-2 (*KDR*) mRNA was expressed at a low level in Cal62 cells, and this expression was significantly downregulated upon EC80 vandetanib treatment (log2FC -2.24, Supplementary Table S1). Since expression of the Annexin V protein in the cells was not significantly altered by vandetanib treatment, this could mean that its secretion as EV cargo was downregulated in order to retain it in the cells as a possible feedback mechanism from the signaling cascade impaired by vandetanib.

Most of the pathways associated with upregulated cellular proteins were linked to protein signaling and cell growth, also matching the functions of the proteins upregulated in all vandetanib doses. However, the pathways associated with downregulated proteins were mainly related to cell cycle progression or DNA damage repair, indicating that although the cellular proliferation pathway proteins were generally upregulated, the cells went into senescence or apoptosis, probably because the activating input from EGFR or VEGFR was missing due to vandetanib inhibition. Interestingly, pathways linked to targets of the aforementioned transcription factor Myc were significantly enriched. Most likely, vandetanib treatment decreased the activity of Myc since the results showed upregulation of genes that are described as downregulated by Myc (e.g., *NDRG1*, *APP*, *ITGB1*), and downregulation of genes described as upregulated by Myc (e.g., *TK1*, *TOP2A*, *UBE2S*)^66,67^. In the EVs, the pathways containing upregulated proteins were associated with apoptosis and energy metabolism, while the pathways with downregulated proteins were mainly linked to cell adhesion and migration, and therefore metastasis. This indicates once more that vandetanib treatment resulted in senescence and decreased viability of Cal62 cells.

Except for the EC50 vandetanib dose and mitomycin C, no significant Spearman correlations were observed between the vandetanib-induced log2FCs and the mean LINCS Z-scores of the three cytotoxic drugs for the eight biomarker genes. These results support the conclusion that the identified biomarker signature reflects a specific transcriptional response to vandetanib, rather than a general cytotoxic effect.

Finally, this study has several limitations that should be acknowledged. First, all experiments were conducted using a single cell line (Cal62) with just three biological replicates. Nonetheless, the low variability observed in the cellular transcriptomic and proteomic data suggests a strong and consistent response to vandetanib treatment, indicating similar results may be obtained with additional replicates or with other ATC cell lines exhibiting EGFR overexpression. Another limitation is the absence of patient-derived samples, which leaves the applicability of the identified biomarker signature in more complex biological matrices such as human plasma to be demonstrated. In plasma, EVs and cfRNA shed by healthy cells are also present and are co-isolated with tumor-derived material. Since RTKs are also expressed in healthy tissues, especially EGFR in epithelial cells, vandetanib treatment also affects those tissues, as evidenced by its known side effects^68^. Nonetheless, tumor cells typically release significantly more EVs than normal cells, suggesting that cancer-derived cfRNA may predominate^69^. Moreover, ATC tissues in patients considered for off-label treatment with vandetanib usually exhibit pathologically high overexpression of vandetanib targets, increasing the likelihood that the drug will preferentially bind to cancer cells rather than healthy tissue. Notably, several prior studies have validated liquid biopsy biomarkers in patient body fluids that were initially identified through in vitro experiments, supporting the potential clinical relevance of our biomarker signature^70–73^.

Despite these limitations, this study is the first to comprehensively investigate the impact of vandetanib on the transcriptome and proteome of ATC cells and propose a biomarker signature for monitoring the therapy success in vitro, offering a valuable foundation for future research and potential clinical translation.

## Conclusion

Given the rapid growth and aggressiveness of ATC, it is of utmost importance to evaluate the therapeutic success as soon as possible. Additionally, off-label therapy is becoming increasingly important in heterogeneous and complex diseases like ATC. In this study, we identified an EV-associated, cell-free transcriptional biomarker signature for the vandetanib in vitro treatment of Cal62 ATC cells. Furthermore, we elucidated the holistic transcriptional and proteomic changes induced in these cells by vandetanib treatment in three different doses. To the best of our knowledge, this is the first published work regarding the treatment of Cal62 cells with vandetanib and the transcriptional and proteomic effects of vandetanib on ATC. Because of the limited number of ATC patients undergoing off-label vandetanib treatment, these results are an important basis for designing further studies involving valuable patient liquid biopsy samples. Our results might also provide helpful information for decision-making in single ATC patients treated with vandetanib since no clinical study results are yet available.

## Methods

### Cell culture and vandetanib treatment

Cal62 cells (RRID:CVCL_1112) were obtained from Prof. Netea-Maier’s lab (Radboudumc, Netherlands) through the EU Horizon Project REPO4EU, and cultured in EV-depleted medium. For EV depletion, the medium – DMEM (Gibco 41,965) without Pyruvate and supplemented with 4.5 g/l D-Glucose and L-Glutamine, 10% fetal calf serum (FCS, Sigma Aldrich F7524, Lot Nr. 0001664377), 5 mM HEPES (Invitrogen 15630056), 50 µM 2-mercaptoethanol (Invitrogen 31350010) and 100 U/ml Penicillin–Streptomycin (Amimed 4-01F00-H) – was supplemented with 20% FCS and ultracentrifuged for 18 h at 100,000 g, as described by Théry et al. in 2006^74^. After ultracentrifugation, the medium was carefully collected from the centrifugal tubes by pipetting, discarding the bottom 10% of the volume to minimize possible FCS EV transfer to the depleted medium. The depleted medium was then mixed with FCS-free medium to an FCS concentration of 10% and sterile-filtered (0.22 µm) before storage.

Vandetanib (Thermo Fisher Scientific 464332500) was dissolved in DMSO to 20 mM and subsequently diluted with EV-depleted medium to the required concentrations: 2.4, 8.8, and 20 µM for the main experiment; and 1.25, 2.5, 5, 7.5, 10, 15, and 20 µM for the viability assay. A DMSO concentration of 0.1% was used as the non-treatment control. The culture medium was used as an additional control for viability assays. Cell seeding density was 5850 live cells per cm^2^ and culture volumes were 100 µl/well (TPP 92,096) or 20 ml/flask (TPP 90,151). The culture medium was carefully removed 24 h after seeding and replaced with medium containing the respective vandetanib / DMSO concentration.

Cell viability was assessed using the EZMTT cell proliferation assay (Sigma Aldrich CBA410) according to the manufacturer’s instructions. The EC10, EC50, and EC80 values were determined using a variable slope four-parameter non-linear model in GraphPad Prism 10 (RRID:SCR_002798). All cell culture experiments were performed in triplicate, and cultures were regularly confirmed negative for mycoplasma contamination by qPCR using the primers described by Young et al. in 2010^75^.

For preliminary cell viability assays (Supplementary Fig. S2), 8505C and Cal62 cells were cultured in DMEM with 10% FCS, 1% GlutaMAX supplement (Gibco 35,050,061), and 1% ml Penicillin–Streptomycin. 2000 cells were seeded per well, and vandetanib treatment was performed 24 h after seeding at 0.3125, 0.625, 1.25, 2.5, 5, and 10 µM, with a DMSO concentration of 0.1% as the non-treatment control. The viability was measured 48 h after treatment using the CellTiter-Glo 2.0 Cell Viability Assay (Promega G9241) according to the manufacturer’s instructions. Dose–response curves were plotted in GraphPad Prism 10.

### EV concentration and characterization

All EV experiments were conducted in accordance with the MISEV guidelines to the best of our ability^50,63,76^. The entire cell culture supernatant was centrifuged at 300 g for 10 min and subsequently at 2,000 g for 10 min to remove any cells, apoptotic bodies and cellular debris. EVs were concentrated from the pre-cleared supernatant by precipitation with 10% v/v 6 kDa polyethylene glycol (PEG-6000). After incubation on ice for 2 h, the precipitated EVs were pelleted by centrifugation at 3,200 g for 30 min and resuspended in 240 µl PBS.

The particle concentration and size distribution of the EV preparation were assessed via NTA using a ZetaView PMX 110 (Particle Metrix, RRID:SCR_016647) equipped with a 520 nm laser. All 11 positions were recorded in two cycles (30 fps, shutter: 70, sensitivity: 85) at 23 °C. The image evaluation settings in the ZetaView Software version 8.05.12 SP1 were adjusted for EV detection (brightness: 20–255, area: 5–1000, trace length: 15, classes/decade: 64, 5 nm/class). More than 500 traced particles per sample were measured.

For FC EV analysis, 5E8 EV particles were first diluted in 200 µL of PBS. A 300 µl antibody master mix was prepared, containing 10 µM carboxyfluorescein succinimidyl ester (CFSE; 1:500 dilution, BioLegend 423801), 2.5 µL of PanEV antibody mix (1:200 dilution), and 5 µL of APC/Fire 750 anti-EGFR antibody (1:100 dilution, AY13 clone, BioLegend Cat# 352926, RRID:AB_2814285). The PanEV antibody mix was prepared with 1 µL each of PE anti-CD63 (H5C6 clone, Cat# 353003, RRID:AB_10896786), PE anti-CD81 (5A6 clone, Cat# 349506, RRID:AB_10645519), and PE anti-CD9 (HI9a clone, Cat# 312105, RRID:AB_2075893) (all from BioLegend). Isotype controls (IgG) without CFSE staining (APC/Fire 750: BioLegend Cat# 400195, RRID:AB_2942001; PE: BioLegend Cat# 400113, RRID:AB_326435) were included as negative controls. 200 µL of EVs were combined with 300 µL of the antibody master mix and incubated for 1 h at 37 °C.

The stained EVs were then analyzed on the BD LSR Fortessa flow cytometer (Becton, Dickinson and Company). EV populations were initially gated using FSC-A and SSC-A to identify vesicle populations. To enhance signal accuracy, CFSE^+^ EVs were further gated based on CFSE and FSC-A signals. Disease-specific EV populations were identified by gating PanEV^+^ and EGFR^+^ EVs, as compared to isotype controls. Percentages for each sample were adjusted by subtracting the corresponding IgG control values to ensure accurate determination of EV purity and to minimize potential artifacts from nonspecific antibody binding.

### Isolation of cellular RNA and cfRNA

For lysis and total RNA isolation, the Monarch Total RNA Miniprep Kit (New England Biolabs T2010S) was used. Cells were lysed with 1.5 ml lysis buffer per sample directly in the cell culture flasks, immediately after removing the supernatant and washing with PBS. 50 µl nuclease-free water was used for elution. Concentration and purity were assessed on a NanoDrop 1000 spectrophotometer (Thermo Fisher Scientific, RRID:SCR_016517).

Total cfRNA was isolated from the EV preparation using the miRNeasy Micro Kit (Qiagen 217084). The eluate was reapplied to the column once to repeat the elution for maximum RNA recovery. Quantitative and qualitative assessment was performed with an RNA 6000 Pico Kit on a Bioanalyzer 2100 automated electrophoresis system (Agilent Technologies 5067–1513, RRID:SCR_018043).

### Total RNA-Seq

Libraries for cellular total RNA-Seq were prepared using the Watchmaker RNA Library Prep Kit with Polaris Depletion (Watchmaker Genomics BK0002-096), with the Illumina universal adapter and index primers from the Twist UDI adapter system (Twist Bioscience 101308) and Mag-Bind TotalPure NGS beads (Omega Bio-Tek M1378) for all bead purification steps. The RNA was fragmented for 5 min at 85 °C, the adapter was diluted to 2 µM before use, and the second post-ligation cleanup was skipped. Library amplification was performed with 12 cycles. A Bioanalyzer DNA High Sensitivity Chip (Agilent Technologies 5067–4626) was used for library quality assessment and quantification. Next-generation sequencing was performed at Novogene Europe GmbH, Munich, on an Illumina NovaSeq X Plus (RRID:SCR_024568) 10B flow cell lane (1.25B reads) with 1% PhiX sequencing control.

The nf-core/rnaseq (https://nf-co.re/rnaseq, version 3.14.0, RRID:SCR_026973) pipeline was used for QC and processing of the raw RNA-Seq FASTQ data in R (https://www.r-project.org/, version 4.4.1, RRID:SCR_001905) using the RStudio application (https://posit.co/download/rstudio-desktop/, version 2024.9.1.394, RRID:SCR_000432)^77–80^. As part of the pipeline, rRNA reads were removed using SortMeRNA (https://sortmerna.readthedocs.io/, version 4.3.4, RRID:SCR_014402)^81^. The remaining reads were mapped to the human genome (GRCh38) using the STAR aligner (https://github.com/alexdobin/STAR, version 2.7.9, RRID:SCR_004463) in combination with the Ensembl genome annotation (https://www.ensembl.org/, version 107, RRID:SCR_006773)^82,83^. Gene expression was quantified with Salmon (https://combine-lab.github.io/salmon/, version 1.10.1, RRID:SCR_017036)^84^. Read count normalization and differential gene expression analysis were performed in R using the *DESeq2* package (10.18129/B9.bioc.DESeq2, version 1.46.0, RRID:SCR_015687)^85^. For dimensional reduction – principal component analysis (PCA) and (sparse) partial least-squares discriminant analysis (s)(PLS-DA) – the R package *mixOmics* (https://mixomics.org/, version 6.30.0, RRID:SCR_016889) was used^86^. Pathway ORA was performed against the Gene Ontology (https://geneontology.org/, GO, RRID:SCR_002811) database including the aspects Molecular Function (MF) and Biological Process (BP), the Molecular Signatures Database (https://www.gsea-msigdb.org/gsea/msigdb, MSigDB, RRID:SCR_016863), the Kyoto Encyclopedia of Genes and Genomes (https://www.kegg.jp/, KEGG, RRID:SCR_012773), the Reactome database (https://reactome.org/, RRID:SCR_003485), and the WikiPathways database (https://www.wikipathways.org/, RRID:SCR_002134)^42–44^. The R packages *clusterProfiler* (10.18129/B9.bioc.clusterProfiler, version 4.14.4, RRID:SCR_016884) and *msigdbr* (https://igordot.github.io/msigdbr/, version 7.5.1, RRID:SCR_022870) were used, and the universe was defined as all genes detected in the cellular RNA-Seq^87,88^.

### Proteomics

Cells were lysed directly in the flasks using 1 ml 1 × RIPA buffer (Abcam ab156034) with 1 × ProteaseArrest protease inhibitor cocktail (G-Biosciences 786–711). A total of 5E8 EVs per sample were prepared in the same buffer. Cellular and EV samples were boiled at 70 °C for 10 min, sonicated at 4 °C for 5 min, and then centrifuged at 10,000 g at 4 °C for 30 min. Protein concentration was determined using a Pierce BCA assay (Thermo Fisher Scientific A55864). Subsequently, 14 µg of EV proteins and 25 µg of cellular proteins were prepared in 4 × Laemmli Sample Buffer (Bio-Rad 1,610,747) containing 2-mercaptoethanol (Merck 805,740) in a final volume of 30 µl. Proteomics sample preparation was performed by in-gel trypsin digestion as described previously^89^.

Proteomics measurements were carried out on a Vanquish™ Neo UHPLC (microflow configuration; Thermo Fisher Scientific, MA, USA; RRID:SCR_026495) coupled to an Orbitrap Exploris 480 mass spectrometer (Thermo Fisher Scientific, MA, USA; RRID:SCR_027000). The workflow was performed as previously published, with minor adjustments^90^. Peptides were applied onto a commercially available Acclaim PepMap 100 C18 column (2 μm particle size, 1 mm ID × 150 mm, 100 Å pore size; Thermo Fisher Scientific, MA, USA) and separated on a stepped gradient from 3 to 31% solvent B (0.1% FA, 3% DMSO in ACN) in solvent A (0.1% FA, 3% DMSO in HPLC grade water) over 60 min. A flow rate of 50 μl/min was applied. The mass spectrometer was operated in DDA and positive ionization mode. MS1 full scans (360 – 1300 m/z) were acquired with a resolution of 60,000, a normalized automatic gain control target value of 100%, and a maximum injection time of 50 ms. Peptide precursor selection for fragmentation was carried out using a cycle time of 1.2 s. Only precursors with charge states from two to six were selected, and dynamic exclusion of 30 s was enabled. Peptide fragmentation was performed using higher energy collision-induced dissociation and a normalized collision energy of 28%. The precursor isolation window width of the quadrupole was set to 1.1 m/z. MS2 spectra were acquired with a resolution of 15,000, a fixed first mass of 100 m/z, a normalized automatic gain control target value of 100%, and a maximum injection time of 40 ms.

Peptide identification and quantification were performed similarly to our prior publication^89^, using the MaxQuant software (https://www.maxquant.org/, version 1.6.3.4, RRID:SCR_014485)^91^, with its built-in search engine Andromeda^92^. MS2 spectra were searched against the human protein database from UniProt (https://www.uniprot.org/, UP000005640, downloaded on May 4th, 2020, RRID:SCR_002380), supplemented with common contaminants (using the built-in option). Trypsin/P was specified as the proteolytic enzyme, and carbamidomethylated cysteine was set as a fixed modification. Oxidation of methionine and acetylation at the protein N-terminus were defined as variable modifications. The results were adjusted to a 1% false discovery rate (FDR) at both the peptide-spectrum match (PSM) and protein levels using a target-decoy approach with reversed protein sequences.

Label-free quantification (LFQ) intensities were further analyzed using Perseus (https://maxquant.net/perseus/, version 2.0.9.0, RRID:SCR_015753)^93^. Perseus’s built-in filter functions were used to remove reverse hits, proteins identified only by site, and contaminants. Only proteins with a peptide count greater than 1 and detected in at least 50% of the samples in at least one group were retained. All proteins detected in the culture medium samples were removed from the EV samples. For protein quantification, LFQ intensities were log10-transformed. Missing values were imputed using a normal distribution with default settings (width = 0.3, down shift = 1.8). The imputed dataset was normalized by quantile normalization before statistical testing. Paired t-tests were conducted on the imputed/normalized datasets, followed by Benjamini–Hochberg correction (FDR = 0.05). Enrichment analysis of significant proteins was performed against terms from the GO, KEGG, GSEA, and Reactome databases using Fisher’s exact test with Benjamini–Hochberg FDR correction and a threshold value of 0.01^42–44^.

### RT-qPCR

Transcriptomic biomarker signature candidates were validated by RT-qPCR. The newest MIQE guidelines were applied while performing all RNA handling and RT-qPCR experiments^94,95^. RNA samples (cellular and EV-cfRNA) were reverse transcribed using the LunaScript RT SuperMix Kit (New England Biolabs E3010). qPCR was performed on a MIC qPCR cycler (Bio Molecular Systems) using the Luna Universal qPCR Master Mix (New England Biolabs M3003), 40 cycles and 0.3 °C/s increments for the melting curve. The primers used for RT-qPCR were either ordered as premixed primer assays from BioRad or designed using Primer3 (https://primer3.ut.ee/, RRID:SCR_003139)^96–98^. All primers used for the RT-qPCR experiments shown in this paper are listed in ( \* MERGEFORMAT Table 3). Primers were used at 1 × concentration (BioRad primers) or 480 nM (Primer3-designed primers).

**Table 3:** Sequences and information of the RT-qPCR primers used for validation of the final eight biomarkers. fw = forward, rv = reverse, bp = base pairs.

Target	Primer sequences (5’—> 3’)	Source	Tm	Amplicon size
ARF1	–	BioRad (qHsaCED0045157)	60 °C	–
UBC	fw: CTGGTGCTCCGTCTTAGAGG rv: TTTCCCAGCAAAAGATCAACC	Designed with Primer3	60 °C	169 bp
GAPDH	fw: CAGCCTCAAGATCATCAGCA rv: GTCTTCTGGGTGGCAGTGAT	Designed with Primer3	58 °C 60 °C	135 bp
NDRG1	fw: GACCTGGAGATTGAGCGACC rv: CCCAACCACCAACAGAGCA	Designed with Primer3	60 °C 60 °C	75 bp
ISG20	fw: GCCTCCTACACAAGAGCATC rv: CCCTCGCATCTTCCACCG	Designed with Primer3	61 °C 60 °C	64 bp
FOXM1	fw: AGCGACAGGTTAAGGTTGAGG rv: TCTCAGTGCTGTTGATGGCG	Designed with Primer3	60 °C 61 °C	120 bp
TPX2	fw: ATGATGCCCCCTCGGATTTC rv: AGCCCTCCAGTTCCATTCTTC	Designed with Primer3	60 °C 60 °C	133 bp
MYBL2	fw: CCTGCCTTACAAGTGGGTGG rv: ACCAAGCATCAGGGTCCGA	Designed with Primer3	61 °C 61 °C	107 bp
AHNAK2	fw: GGAAGCGAGATTCAGGGGAC rv: GGACAGCCTCTGGACGACT	Designed with Primer3	60 °C 61 °C	60 bp
GPRC5C	fw: GATGCACAAAGTTCCGTCCG rv: AGTACATGTCTTCAGCCCGC	Designed with Primer3	60 °C 60 °C	113 bp
THBD	fw: GGGGTGATTAGAGGGAGGAGA rv: TTGCCCAGTGGTCCAGTGA	Designed with Primer3	60 °C 61 °C	65 bp

ARF1, UBC and GAPDH were chosen as reference genes for the cell-free RNA, and UBC and GAPDH for the cellular RNA after analyzing Cq values of multiple reference gene candidates from a preliminary Cal62 vandetanib treatment EV experiment with the algorithms NormFinder (https://www.moma.dk/software/normfinder, RRID:SCR_003387)^99^ and geNorm (https://genorm.cmgg.be/, RRID:SCR_006763)^100^. Log2FCs were calculated by normalizing the Cq values to the mean Cq of the reference genes and with respect to the PCR amplification efficiency^101^.

## Supplementary Information

Supplementary Information.

## Data Availability

The RNA-Seq data were uploaded to the European Nucleotide Archive under accession number PRJEB88458. The Proteomics data files have been deposited to the ProteomeXchange Consortium via the PRIDE partner repository and can be accessed using the identifier PXD064007. RT‐qPCR data are available on Zenodo (10.5281/zenodo.15425227). The R code used for RNA-Seq data evaluation (after gene expression quantification with Salmon) was uploaded to GitHub (https://doi.org/10.5281/zenodo.16914565).
