# Supplementary figures and images for: Extracellular vesicle-associated transcriptomic and proteomic biomarkers show in vitro potential for vandetanib treatment monitoring in anaplastic thyroid cancer
Source: Sci Rep. 2025 Sep 12;15:32464. doi: 10.1038/s41598-025-18319-w (PMC12432113; doi:10.1038/s41598-025-18319-w)

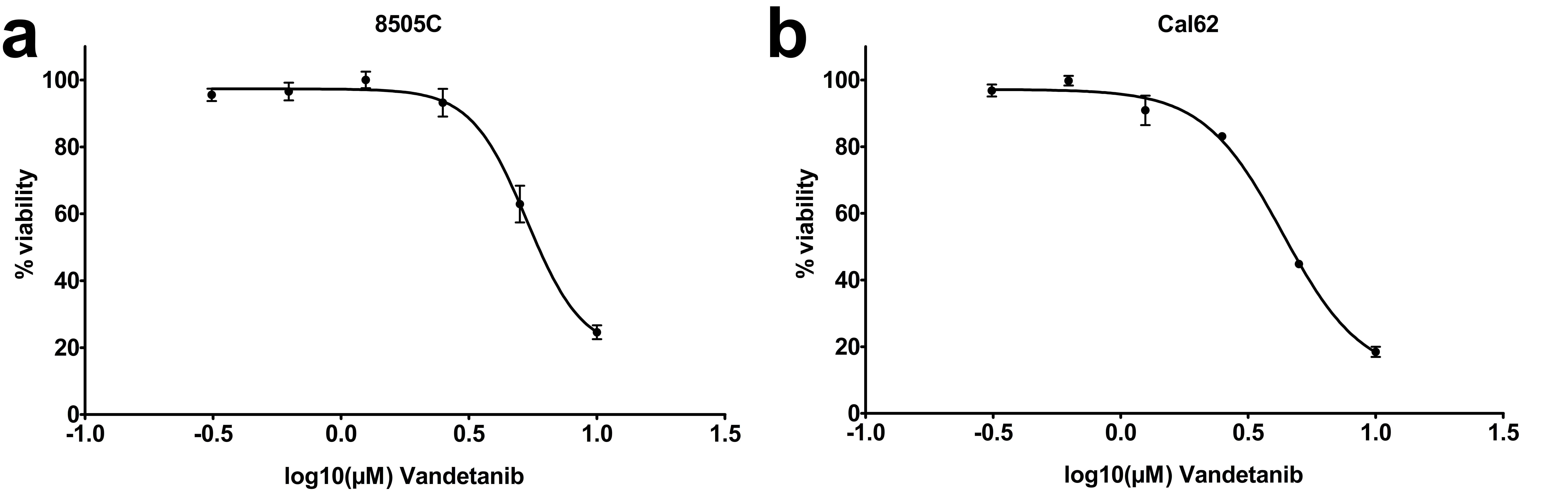

Supplement: Supplementary file 1 — Supplementary Information. [file 41598_2025_18319_MOESM1_ESM.zip › Supplementary Figure S1.png]
